# LncRNA ROR/miR-145-5p axis modulates the osteoblasts proliferation and apoptosis in osteoporosis

**DOI:** 10.1080/21655979.2021.1982323

**Published:** 2021-10-07

**Authors:** Yin Fu, Xiaoyang Hu, Yanyu Gao, Kai Li, Qiang Fu, Qingpeng Liu, Dan Liu, Zhijia Zhang, Jiutao Qiao

**Affiliations:** a Department of Basic Medical Sciences, Heilongjiang University of Chinese Medicine, Harbin, China; b Department of Chinese Formulae, Heilongjiang University of Chinese Medicine, Harbin, China; c Department of Interventional Radiology, Harbin Medical University Cancer Hospital, Harbin, China; d Department of Orthopedic Surgery, The Second Affiliated Hospital of Harbin Medical University, Harbin, China; e Brown University RI Hospital Liver Research Center, Providence, RI, USA; f Beijing University of Chinese Medicine, Beijing, China

**Keywords:** Osteoporosis, LncRNA ROR, MiR-145-5p, MC3T3-E1

## Abstract

Osteoporosis (OP) is a systemic bone metabolic disease. Promotion of osteoblast proliferation and inhibition of cell apoptosis may be helpful for the prevention and clinical treatment of OP. In the current study, we focused on the expression changes and clinical values of lncRNA ROR and miR-145-5p in OP clinical serum samples, and investigated the interactive modulation effect of ROR/miR-145-5p on osteoblast function. Serum samples were obtained from 82 OP patients and 79 healthy individuals. MC3T3-E1 was applied for the cell experiments. Levels of lncRNA ROR and miR-145-5p were detected using qRT-PCR. Transient transfection was performed to regulate gene levels in cells, and cell proliferation and apoptosis were detected. A reciprocal correlation between lncRNA ROR and miR-145-5p was explored. LncRNA ROR was downregulated, and miR-145-5p was overexpressed in OP patients. The combined diagnosis of ROR and miR-145-5p showed good diagnostic value for OP. ROR knockdown promoted the MC3T3-E1 cell apoptosis and inhibited cell proliferation. Luciferase reporting assay verified the target relationship between ROR and miR-145-5p. MiR-145-5p downregulation reversed ROR silence mediated effect on MC3T3-E1 cell proliferation and apoptosis. LncRNA ROR is downregulated and miR-145-5p is highly expressed in OP patients. ROR knockdown may inhibit osteoblast proliferation via targeting miR-145-5p. It may provide a theoretical basis and experimental basis for ROR to be a potential target for the treatment of OP.

## Background

Osteoporosis (OP), a systemic bone metabolic disease, is characterized by decreased bone mass, decreased bone density, and degeneration of bone microstructure [1]. OP tends to occur in the elderly [2]. Over the past 30 years, the number of cases suffering from OP in China has tripled to 90 million, which is the highest in the world [3]. Because of its slow onset and no specific symptoms in clinical practice, OP is always overlooked [4]. Thus, it is necessary to identify new and sensitive markers for the early diagnosis of OP. At present, the treatment of OP can be divided into three categories: basic drugs (VitD, calcium), anti-absorption drugs and osteogenic drugs [5]. Although these drugs are effective for OP, the first two types cannot induce bone formation and have significant side effects [6]. PTH 1–34 (parathyroid hormone 1–34) can stimulate osteogenesis, but it faces problems of receptor saturation and disorder of PTH secretory axis [7]. Therefore, exploring the mechanism of OP can provide a new method for the treatment or relief of OP.

Accumulating studies have indicated the influence of long noncoding RNAs (lncRNAs) on the progression of many diseases [8]. LncRNAs are non-coding RNAs with a length larger than 200 nucleotides. LncRNA can regulate a variety of cells, including osteoblasts (OB), osteoclasts (OC) and bone marrow mesenchymal stem cells (BMSCs), thereby affecting the process of bone metabolism [9]. The lncRNA regulator of reprogramming (ROR) is recently discovered and has been reported to function in a variety of stem cells, an elevated level of ROR has been detected in embryonic stem cells (ESCs) [10]. The regulatory role of ROR in osteoarthritis (OA) is also reported by Feng et al., overexpression of ROR can promote BMSCs chondrogenesis differentiation and cartilage formation [11]. During the course of bone metabolism, a novel role of ROR is defined to promote osteoblastic differentiation of human mesenchymal stem cells [12]. However, the role of lncRNA ROR has not been examined in the progression of OP.

Recent studies have shown that lncRNAs can serve as competing endogenous RNAs (ceRNAs) and interact with microRNAs (miRNAs). MiRNAs are small endogenous non-coding single stranded RNAs that play a role via targeting to the 3‘-untranslated region (3ʹ-UTR) of the target gene and regulate its levels at the post-transcriptional level [5]. It is widely accepted that miRNAs can regulate various signaling pathways or transcription factors related to osteoblast differentiation [13,14]. Recent studies have also demonstrated that ROR serves as a sponge of miR-145-5p and is involved in the development of several diseases via interacting with miR-145-5p, such as ovarian cancer, chronic hypoxic heart disease [15,16]. In addition, miR-145-5p also plays an important role in the osteocyte function [17]. In a study of postmenopausal osteoporosis, miR-145-5p is demonstrated to be suppressed by estrogen and further promoted the production of osteoprotegerin [17]. The elevated level of miR-145-5p is also detected in both in vitro cell and in vivo mouse models of glucocorticoid‑induced osteoporosis [18]. Therefore, whether miR-145-5p is involved in the regulation mechanism of ROR in OP attracts our interest.

In the current study, the levels of ROR and miR-145-5p in patients with OP were detected, and their diagnostic value was further analyzed. In addition, the effects of ROR and miR-145-5p on the proliferation and apoptosis of osteoblasts were also investigated. We attempt to provide the theoretical and experimental basis for potential therapeutic targets of OP.

## Methods

### Clinical samples

A total of 82 patients with OP who were treated in the Department of Orthopedics of Heilongjiang University of Chinese Medicine from February 2018 to October 2020 were collected as the observation group. In addition, 79 healthy individuals who get a regular physical examination were selected as the control group. The dual-energy X-Ray absorptiometry (DEXA) method was applied for the detection of bone mineral density (BMD). The diagnosis of OP patients was based on the femoral neck and lumbar spine T score (less than −2.5), when the T score of the femoral neck and/or lumbar spine was less than −2.5, OP was finally diagnosed [19]. All participants have a certain level of education and can cooperate with the completion of the research.

This study was approved by the Research Ethics Committee of the second affiliated hospital at Harbin Medical University. The methodology conforms to the standards set out in the Declaration of Helsinki. All the patients and their immediate family members were informed of the study design, and all of them signed the informed consent. Individuals with other orthopedic diseases other than osteoporosis, diabetes, other major organic diseases, and mental illnesses were excluded.

5 mL peripheral venous blood under fasting condition was collected from the patients in the morning, and the blood samples were left standing for 30 min and centrifuged to collect the serum samples.

### Cell culture

Murine osteoblastic cell-line MC3T3-E1 was cultured in α-MEM medium containing 10% fetal bovine serum (FBS) and 100 U/ml penicillin. Cells were inoculated in 25 cm^2^ cell culture flask and incubated in an incubator under the culture temperature of 37°C, containing 5% CO_2_ and 95% relative humidity. After being cultured for 24 h, the cells were observed under an inverted light microscope (Nikon Company, Japan). When the confluence of the cells reached 80–90%, the cells were digested with trypsin for subsequent experiments.

### Cell transfection

The overexpression vector of lncRNA ROR (pcDNA 3.1-ROR), small interfering RNA targeting ROR (si-ROR), miR-145-5p mimic, miR-145-5p inhibitor, the negative control groups (pcDNA 3.1-NC, si-NC miR-NC), were designed and amplified by the RiboBio Corporation (Guangzhou, China). For lncRNA-ROR overexpression, the lncRNA-ROR gene was cloned into the lentiviral-vector pcDNA3.1 (System Biosciences, USA) and primer sequences are as follows: lncRNA-ROR: forward, 5′-GGGGTACCGTTCTCATTTTTCTACTGCTCGTG-3′ and reverse, 5′-CGGGATCCATGTAATCAATCATTTTATTATTTTCATC-3′. The si-NC and si-ROR sequences are below: si-NC: UUCUCCGAACGUGUCACGU; si-ROR: GGAGA GGAAGCCUGAGAGU. The sequences of miR-145-5p mimics and miR-145-5p inhibitors were 5ʹ-GUCCAGUUUUCCCAGGAAUCCCUGGAUUCCUGGGAAAACUGGACUU-3ʹ and 5ʹ-AGGGAUUCCUGGGAAAACUGGAC-3ʹ, respectively, and that of the negative control (miR-NC) was 5ʹ-UUCUCCGAACGUGUCACGUTT-3ʹ. MC3T3-E1 cells were inoculated in 96-well plates, when the degree of confluence reached 50%–60%, 50 nM pcDNA 3.1-ROR, si-ROR, miR-145-5p mimic, miR-145-5p inhibitor or pcDNA 3.1-NC, si-NC or miR-NC were transfected into the cells using Lipofectamine 3000 (ThermoFisher Scientific). 6 h after transfection, fresh culture medium containing 10% FBS was replaced to maintain normal growth of cells. Follow-up experiments were conducted 24 h after transfection.

### qRT-PCR

Total RNA was extracted by Trizol, and its concentration and purity were determined. RNA was reverse transcribed into cDNA using a reverse transcriptometer according to the instructions of the reverse transcription kit. The cDNA was used as a template for qRT-PCR, and the reaction conditions were 95°C for 5 min, 95°C for 15 s, 60°C for 30 s, and 72°C for 30 s, with a total of 40 cycles. GAPDH was used as the reference for lncRNA ROR and U6 was used for miR-145-5p. The levels of ROR and miR-145-5p were calculated using 2^−ΔΔCt^ [20]. The following PCR primers were used: ROR, forward, 5ʹ-AGGAAGCCTGAGAGTTGGC-3ʹ, reverse, 5ʹ- CTCAGTGGGGAAGACTCCAG-3ʹ; GAPDH, forward, 5ʹ-TGTTCGTCATGGGTGTGAAC-3ʹ; reverse, 5ʹ-ATGGCATGGACTGTGGTCAT-3ʹ; miR-145-5p, forward, TGTCCAGTTTTCCCAGGAATC; reverse, CTCAACTGGTGTCGTGGAGTC; U6, forward, CCT GCTTCGGCAGCACAT, U6, reverse, AACGCTTC ACGAATTTGCGT.

### CCK-8 assay

MC3T3-E1 cells were seeded into 96-well plates at a density of 4 × 10^3^ cells/well. To detect the cell viability, 10 μL of CCK-8 was added into each well at 0 h, 24 h, 48 h and 72 h. After the mixture was fully mixed, the cells were placed in a cell incubator in the dark and incubated for 1 h. The absorbance values of each group of cells were detected by enzyme plate analyzer, and the cell proliferation capacity was calculated.

### Flow cytometry assay

MC3T3-E1 cells were collected from each group and washed with PBS twice. Annexin V-FITC (10 μl) and propidium iodide (PI, 5 μl) were added to each group, then mixed and reacted for 15 min at room temperature to avoid light. The apoptotic cells were detected by flow cytometry. Final apoptotic cell number was estimated as a total percentage of early apoptotic cells staining positive for Annexin V and negative for PI and late apoptotic cells positive for both Annexin V and PI. The cell apoptosis rate was represented as the percentage of apoptotic cells in the total number of cells.

### Target gene prediction and luciferase reporting assay

The binding sites of ROR and ROCK1 with miR-145-5p were predicted using STARBASE, an online bioinformatics software. The wild-type (WT) and mutated (MUT) ROR or Rho-associated kinases 1 (ROCK1)-dual luciferase reporter vectors were constructed, and co-transfected into MC3T3-E1 cells with miR-145-5p mimic or inhibitor, respectively. After incubation for 48 hours, the luciferase activity was tested by dual luciferase activity detection kit (Promega, USA).

### Statistical analysis

The experimental data were analyzed by SPSS21.0 software, and the measurement data were expressed by mean ± standard deviation (SD). Each experiment was repeated at least three times, using triplicate parallel samples within each experiment. The statistical outlier limits were calculated (greater than two standard deviations from the mean), and no significant outliers were detected. T-test was used for comparison between two groups, and one-way analysis of variance (ANOVA) was used for comparison between multiple groups. The correlation of different indicators was calculated through Pearson’s correlation analysis. The receiver operating characteristic (ROC) curve was drawn to reflect the diagnostic ability of indicators. *P* < 0.05 was considered statistically significant.

## Results

### Clinical data of the clinical subjects

The clinical characteristics of the study subjects were recorded. As shown in Table 1, among 82 OP patients, there were 22 males and 60 females, with the mean age of 50.48 ± 3.5 years old. Twenty-one males and 58 females constituted the control group, with the mean age of 49.68 ± 4.17 years old. The two groups were matched in terms of age, sex and BMI, with no significant differences (*P* > 0.05). The BMD levels at lumbar spine, femoral neck bone, and total hip bone were lower in the OP group than that in the control group, and the difference was statistically significant (*P* < 0.001).

**Table 1.** Clinical data of the study population

Factors	Control (n = 79)	OP patients (n = 82)	*P* value
Age (years)	49.68 ± 4.17	50.48 ± 3.5	0.193
Gender (male/female)	21/58	22/60	0.972
BMI (kg/m^2^)	23.88 ± 3.04	24.05 ± 3.04	0.176
Lumbar spine bone mineral density (g/cm^2^)	0.90 ± 0.05	0.80 ± 0.03	<0.001
Femoral neck bone mineral density (g/cm^2^)	0.71 ± 0.04	0.64 ± 0.04	<0.001
Total hip bone mineral density (g/cm^2^)	0.75 ± 0.04	0.67 ± 0.03	<0.001

OP: osteoporosis; BMI, body mass index; data are expressed as n or mean ± standard deviation. Differences between groups were compared using Student’s t test for continuous variables, and chi-squared test for categorical variables.

The correlation between ROR and clinical characteristics was also detected using Pearson’s correlation analysis. As illustrated in Table 2, significantly negative associations were observed of serum ROR levels with BMD levels at lumbar spine (r = −0.369, *P* < 0.001), femoral neck bone (r = −0.351, *P* < 0.001), and total hip bone (r = −0.465, *P* < 0.001) in OP patients.

**Table 2.** Correlation between lncRNA ROR and clinical characteristics

Characteristics	Correlation with lncRNA ROR (r)	*P-value*
Age (years)	0.121	0.278
Gender (male/female)	0.097	0.388
BMI (kg/m^2^)	0.075	0.505
Lumbar spine bone mineral density (g/cm^2^)	−0.369	0.001
Femoral neck bone mineral density (g/cm^2^)	−0.351	0.001
Total hip bone mineral density (g/cm^2^)	−0.465	<0.001

BMI, body mass index. The correlation of different indicators was calculated through Pearson’s correlation analysis.

### Levels of lncRNA ROR and miR-145-5p in serum samples of OP patients

**Figure 1.**
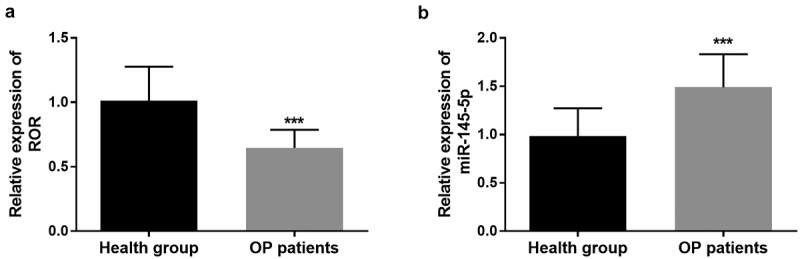
(a) lncRNA ROR levels in the serum of OP patients and healthy controls using qRT-PCR. (b) Levels of miR-145-5p in the serum of OP patients in comparison with the control group. *** *P* < 0.001. Differences between groups were compared using student’s t test

qRT-PCR was applied to detect the level of ROR and miR-145-5p in the serum of study population. Compared with the control group, lncRNA ROR was significantly low expressed in the serum samples of OP group (*P* < 0.001, Figure 1a). However, high levels of miR-145-5p were detected in the serum of OP patients in comparison with the control group (*P* < 0.001, Figure 1b).

### Clinical value evaluation of serum ROR and miR-145-5p for OP diagnosis

**Figure 2.**
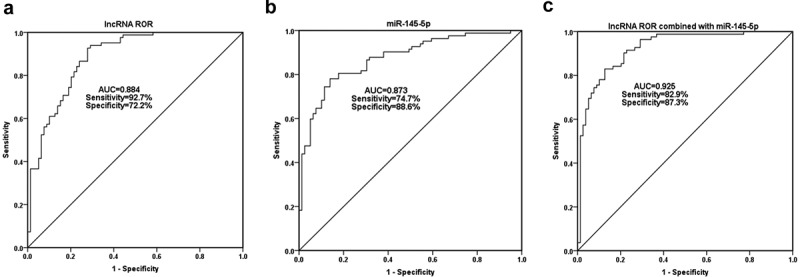
The diagnostic value of serum ROR (a) and miR-145-5p (b) for OP. The AUC of the combined diagnosis of ROR and miR-145-5p was 0.925 (c), which is higher than the diagnostic value of a single indicator

Serum ROR and miR-145-5p levels were included in the ROC analysis. As shown in Figure 2a, the area under the curve (AUC) of serum ROR for OP diagnosis was 0.884, with the sensitivity of 92.7% and the specificity of 72.2% at the cutoff value of 0.816. Serum miR-145-5p also had the diagnostic value for OP, with the AUC of 0.873 at the cutoff value of 1.283 (Figure 2b). As can be seen from Figure 2c, the AUC of combined diagnosis of ROR and miR-145-5p was 0.925, which is higher than the diagnostic value of a single indicator.

### Influence of ROR on MC3T3-E1 cell proliferation and apoptosis

**Figure 3.**
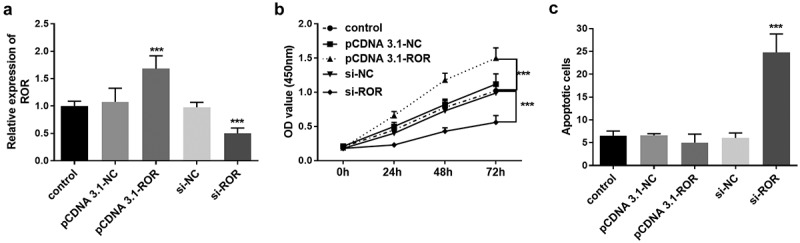
(a) The levels of ROR in different MC3T3-E1 cell groups. (b) The cell viability assessment based on CCK-8 assay. (c) Cell apoptosis evaluation using flow cytometry assay. *** *P* < 0.001, compared with control group. Differences among groups were compared using one-way ANOVA

Considering the dysregulation of ROR in the serum of OP patients, we further explored its role in osteoblast proliferation and apoptosis. The regulation of lncRNA ROR levels in MC3T3-E1 cells was fulfilled by transient transfection of si-ROR and pcDNA-ROR, and significant differences were achieved after transfection (Figure 3a). At different time points after transient transfection, cell viability was detected using CCK-8. The cell viability was promoted significantly when the level of ROR was overexpressed, in contrast, the cell viability was remarkably inhibited by ROR knockdown (Figure 3b). A similar effect was also detected in cell apoptosis. Flow cytometry assay showed a low apoptotic rate in the cell group transfected with pcDNA-ROR, although the difference was not statistically significant (Figure 3c). Conversely, ROR knockdown promoted the MC3T3-E1 cell apoptosis significantly compared with the control group (Figure 3c).

### Reciprocal correlation between lncRNA ROR and miR-145-5p

**Figure 4.**
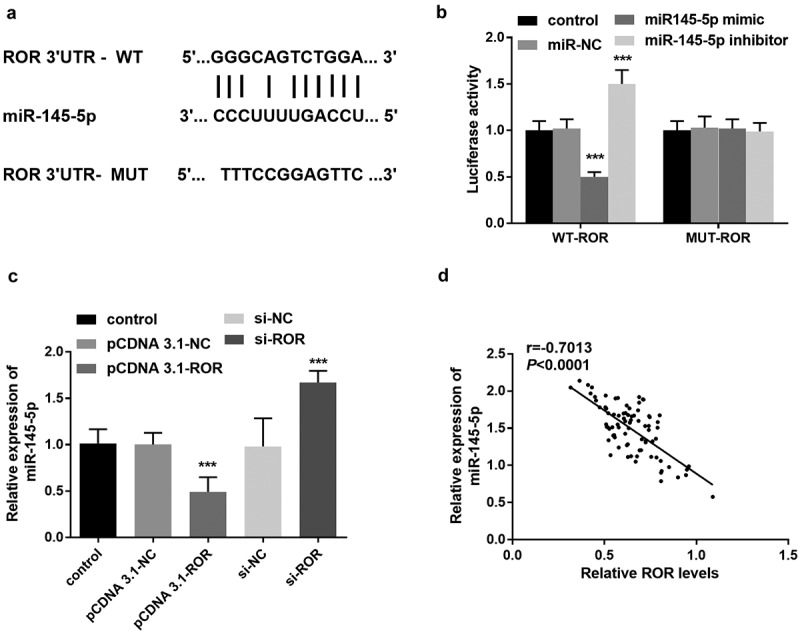
(a) STARBASE predicted the binding sites between lncRNA ROR and miR-145-5p. (b) The luciferase activity of cells transfected with WT-ROR vector or MUT ROR vector and miR-145-5p mimic, or inhibitor. *** *P* < 0.001, compared with control group. (c) Levels of miR-145-5p in different MC3T3-E1 cell groups. *** *P* < 0.001, compared with control group. (d) Association between serum ROR and miR-145-5p levels. Differences among groups were compared using one-way ANOVA

LncRNAs recognize complementary sequences and can bind to miRNAs in a targeted manner. By using the online STARBASE, the binding sites between lncRNA ROR and miR-145-5p were predicted, and illustrated in Figure 4a. To verify the target correlation between ORRO and miR-145-5p, luciferase reporting assay was performed. Based on the results, the luciferase activity of WT-ROR vector was reduced significantly after transfection with miR-145-5p mimic, but the luciferase activity was elevated after the miR-145-5p inhibitor was transfected into MC3T3-E1 cells (Figure 4b). According to the qRT-PCR results, in MC3T3-E1 cells transfected with si-ROR, the level of miR-145-5p was significantly elevated; however, ROR overexpression led to the downregulation of miR-145-5p (Figure 4c). Clinically, there was a significantly inverse association of serum ROR level with miR-145-5p level in OP patients (r = −0.7013, *P* < 0.0001, Figure 4d).

### MiR-145-5p downregulation reversed ROR silence mediated effect on MC3T3-E1 cell behaviors

**Figure 5.**
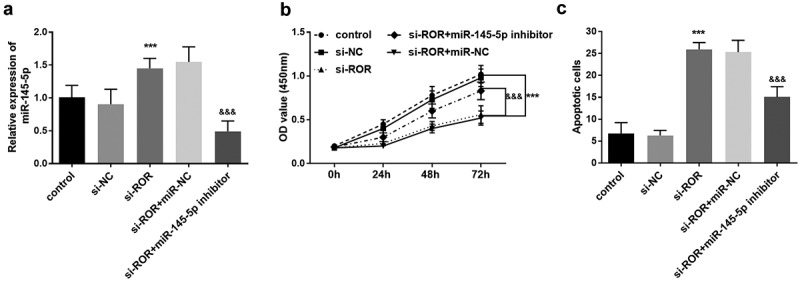
(a) qRT-PCR results for the measurement of miR-145-5p levels in different cell groups. (b) CCK-8 assay results reflecting the cell proliferation in different groups. (c) Cell apoptosis evaluation using flow cytometry assay. *** *P* < 0.001, compared with control group; ^&&&^ *P* < 0.001, compared with si-ROR group. Differences among groups were compared using one-way ANOVA

The involvement and role of miR-145-5p in MC3T3-E1 cell function was also explored. Compared with the control group, si-ROR transfection contributed to the increase of miR-145-5p level, in contrast, after transfected with miR-145-5p inhibitor, the increased trend of miR-145-5p level induced by si-ROR was reversed (Figure 5a). The CCK-8 assay demonstrated that ROR silence significantly inhibited the MC3T3-E1 cell proliferation (Figure 5b). After transfected with miR-145-5p inhibitor, ROR silence mediated cell viability inhibition was significantly rescued (Figure 5b). The cell apoptosis was promoted remarkably following ROR silencing, which was abrogated by miR-145-5p inhibitor transfection (Figure 5c).

### ROCK1 is the target gene of miR-145-5p

**Figure 6.**
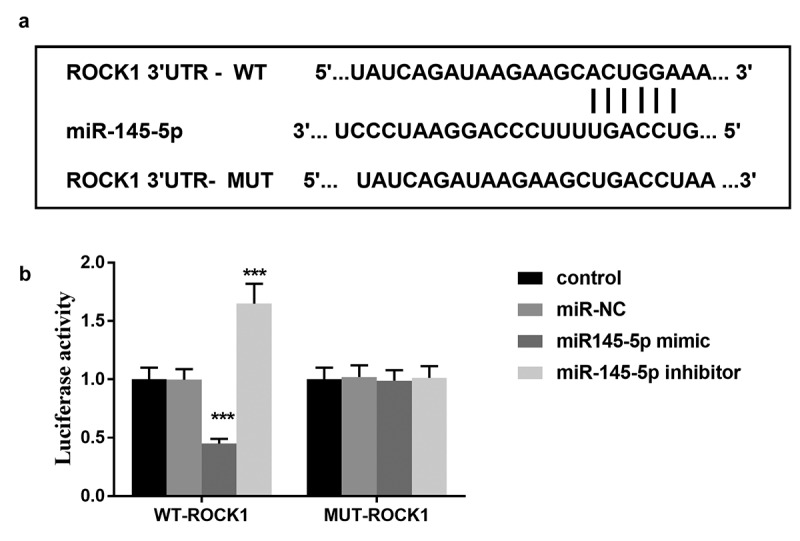
(a) Bioinformatics analysis showed that miR-145-5p contains binding sites for ROCK1. (b) The luciferase activity of different cell groups. *** *P* < 0.001, compared with control group

Bioinformatics analysis showed that miR-145-5p contains binding sites for ROCK1 (Figure 6a). Furthermore, the luciferase reporter assay results demonstrated that transfection of miR-145-5p mimic decreased the luciferase activity in cells transfected with wild-type 3ʹ-UTR of ROCK1, whereas the luciferase activity was increased by miR-145-5p inhibitor transfection significantly (Figure 6b). However, the mutation in the miR-145-5p binding sites in the 3ʹ-UTR of ROCK1 abolished the effect on the luciferase activity (Figure 6b). The results indicated that ROCK1 is a direct target gene of miR-145-5p.

## Discussion

With the development of the social economy and the aging of the population, OP has become a major disease affecting human health. The main pathological features of OP are increased osteoclast activity and reduced osteoblast generation [21]. Osteoblasts are special terminal differentiation products of mesenchymal stem cells, which play an important role in bone development and bone metabolism and contribute to bone formation [22]. Promotion of osteoblasts proliferation and inhibition of cell apoptosis may be helpful for the prevention and clinical treatment of OP. In the present study, we focused on the expression changes and clinical values of lncRNA ROR and miR-145-5p in OP clinical serum samples, and investigated the interact modulation effect of ROR/miR-145-5p on osteoblasts proliferation and apoptosis.

At present, the common clinical diagnosis of OP is based on the BMD values using imaging means [23]. This method is intuitive, noninvasive and safe, and has been widely used in clinical practice. However, due to its difficulty in accurately measuring the changes of early OP and insufficient dynamic monitoring of bone metabolism, this method has some limitations [24]. In the present study, because of deregulated expression of lncRNA ROR and miR-145-5p, ROC curves were established for the measurement of their diagnostic values. As expected, the AUC of both serum ROR and miR-145-5p was all greater than 0.6, and the AUC of the combined diagnosis was the largest. The combined diagnosis of ROR and miR-145-5p has good diagnostic value and can be used as one of the promising reference bases to assist in the diagnosis of osteoporosis in clinical practice.

LncRNA ROR is recently discovered, and the effects and functions of ROR have been widely studied in various cancers, such as gastric cancer, liver cancer, prostate cancer [25–27]. In the present study, we defined a novel role of lncRNA ROR in OP, a low level of ROR was detected in the clinical serum samples of OP patients. Of note, a negative correlation was also detected between ROR level and BMD levels in OP patients. Based on the clinical results, we speculated that lncRNA ROR might play a role in the progression of OP. As previously reported, ROR mediates the osteoblastic differentiation ability of human mesenchymal stem cells, and thus participating in bone metabolism [12]. In addition, upregulated ROR is also demonstrated to promote BMSCs chondrogenesis and cartilage formation activities [11]. These results provide evidence supporting the key role of lncRNA ROR in OP. Furthermore, the regulatory effects of ROR on cell proliferation and apoptosis were further verified in MC3T3-E1 cell lines. Results of the in vitro experiments indicated that ROR knockdown significantly inhibited osteoblast proliferation and promoted cell apoptosis. The results confirmed what we suspected.

It is widely accepted that lncRNAs recognize complementary sequences and can bind to miRNAs in a targeted manner. With the in-depth research on miRNAs, more and more evidence show that miRNAs are closely related to the pathogenesis and progression of OP [28,29]. In the present study, miR-145-5p was identified to be a potential target of ROR, and the luciferase activity assay confirmed the binding relationship between ROR and miR-145-5p. Moreover, the level of miR-145-5p was downregulated by ROR overexpression in MC3T3-E1 cell lines. In a study about postmenopausal osteoporosis, miR-145-5p is regarded to promote the production of osteoprotegerin, and a low level of miR-145-5p is detected in postmenopausal osteoporosis, which supported our findings in the OP clinical samples. In addition, an elevated level of miR-145-5p is also detected in both in vitro cell and in vivo mouse models of glucocorticoid‑induced osteoporosis, which was consistent with our present results [18]. In addition, the rescue experiments in the present study also demonstrated that after transfected with miR-145-5p inhibitor, si-ROR mediated inhibition of osteoblast proliferation and promotion of osteoblast apoptosis was remarkably reversed. Consequently, we hypotheses that lncRNA ROR/miR-145-5p may relate to the osteoblast proliferation in OP.

ROCK1, as one of the most extensively studied RhoA effector proteins, is a key regulator of actin cytoskeleton and cell polarity . Overexpression of R [30]OCK1 has been reported to promote osteoblast viability and inhibit osteoblast apoptosis [31]. The elevated level of ROCK1 is determined to be involved in the anti-osteoporosis effect of Strontium ranelate (SrR) [31]. It is considered to be a therapeutic target for osteoporosis [32]. In the present study, ROCK1 is identified to be the target gene of miR-145-5p in MC3T3-E1 cells. In light of the previous evidence, we deduced that ROCK1 might be involved in the role of ROR/miR-145-5p in osteoporosis. However, it is necessary to carry out further investigation exploring the underlying mechanisms in the future research. In addition, the MC3T3-E1 cell is selected for the cell experiments, which is a mouse osteoblast cell line. In the future, cell experiments performed in a human osteoblast cell line will be necessary to verify the present results.

## Conclusions

Collectively, the study demonstrated that lncRNA ROR is downregulated and miR-145-5p is highly expressed in OP patients. The combined diagnosis of ROR and miR-145-5p has good diagnostic value and can be used as one of the reference bases to assist in the diagnosis of osteoporosis in clinical practice. ROR knockdown may inhibit osteoblast proliferation via targeting miR-145-5p. It may provide a theoretical basis and experimental basis for ROR to be a potential target for the treatment of OP.

## Data Availability

The data that support the findings of this study are available from the corresponding author upon reasonable request.

## Highlights

LncRNA ROR is downregulated and miR-145-5p is highly expressed in OP patients.

ROR knockdown may inhibit osteoblast proliferation via targeting miR-145-5p.

ROCK1 might be involved in the role of ROR/miR-145-5p in osteoporosis.
